# Varied hypoxia adaptation patterns of embryonic brain at different development stages between Tibetan and Dwarf laying chickens

**DOI:** 10.1186/s12864-023-09457-4

**Published:** 2023-06-21

**Authors:** Qiguo Tang, Runjie Yu, Yubei Wang, Fuyin Xie, Hao Zhang, Changxin Wu, Meiying Fang

**Affiliations:** 1 State Key Laboratory of Agrobiotechnology, College of Biological Sciences, China Agricultural University, Beijing, China; 2 Department of Animal Genetics and Breeding, National Engineering Laboratory for Animal Breeding, MOA Laboratory of Animal Genetics and Breeding, College of Animal Science and Technology, China Agricultural University, No.2 Yuanmingyuan West Road, Beijing, 100193 China

**Keywords:** Tibetan chickens, RNA profile, Metabolome, Embryonic brain, Hypoxia

## Abstract

**Background:**

Tibetan chickens (*Gallus gallus*; TBCs), an indigenous breed distributed in the Qinghai-Tibet Plateau, are well adapted to the hypoxic environment. Currently, the molecular genetic basis of hypoxia adaptation in TBCs remains unclear. This study investigated hypoxia adaptation patterns of embryonic brain at different development stages by integrating analysis of the transcriptome with our previously published metabolome data in TBCs and Dwarf Laying Chickens (DLCs), a lowland chicken breed.

**Results:**

During hypoxia, the results revealed that 1334, 578, and 417 differentially expressed genes (DEGs) (|log_2_ fold change|>1, p-value < 0.05) on days 8, 12, and 18 of development, respectively between TBCs and DLCs. Gene Ontology (GO) and pathway analyses revealed that DEGs are mainly related to metabolic pathways, vessel development, and immune response under hypoxia. This is consistent with our metabolome data that TBCs have higher energy metabolism than DLCs during hypoxia. Some vital DEGs between TBCs and DLCs, such as *EPAS1, VEGFD, FBP1, FBLN5, LDHA*, and *IL-6* which are involved in the HIF pathway and hypoxia regulation.

**Conclusion:**

These results suggest varied adaptation patterns between TBCs and DLCs under hypoxia. Our study provides a basis for uncovering the molecular regulation mechanism of hypoxia adaptation in TBCs and a potential application of hypoxia adaptation research for other animals living on the Qinghai-Tibet Plateau, and may even contribute to the study of brain diseases caused by hypoxia.

**Supplementary Information:**

The online version contains supplementary material available at 10.1186/s12864-023-09457-4.

## Introduction

Tibetan chickens (*Gallus gallus*; TBCs), a unique plateau chicken breed, have a very wide distribution at altitudes of 2,200–4,100 m in the Qinghai-Tibet Plateau and have been present in the region for at least 1,000 years [1]. Therefore, they are a good model for studying adaptation to hypoxia. Through a long period of natural and artificial selection, the body appearance and physiology of TBCs have changed compared with lowland chickens to adapt to extreme environments such as high altitude, low oxygen, and cold. TBCs have a smaller size and stronger blood oxygen transport capacity because of more red blood cells (RBCs), lower arterial oxygen partial pressure, lower venous blood pH, and higher hemoglobin concentration [1–3].

Oxygen is essential for regulating tissues and cells to maintain healthy cellular and systemic functions. Hypoxia has important effects on biological activity in animals, and cells in this state are unable to maintain basic life activities and suffer a variety of serious problems [4, 5]. For chickens, the oxygen concentration is a critical factor affecting hatching success, and increases during incubation can dramatically improve hatchability [6–8]. In avian species, hypoxia during incubation inhibits embryonic development and impairs the development of some organs, especially the brain [9–12]. The avian brain is an organ that develops earlier at the embryonic stage and the development of the brain plays a key role in the development of the entire embryo [13]. The oxygen consumed in the chicken brain has not been accurately studied, but in adults, the brain accounts for approximately 2% of the body weight but consumes 20% of the total oxygen consumed by the body under normoxia [14, 15]. As the highest nerve center and most oxygen-sensitive organ, it is important to understand changes in gene expression in the brain under hypoxic incubation conditions, especially in the study of adaptation to high-altitude hypoxia.

Many studies on the hypoxia adaptation mechanism of indigenous animals in the Qinghai-Tibet Plateau have been carried out using transcriptome and genomic analyses, and related progress has mainly involved energy metabolism, hypoxia response, the Ca^2+^ signaling pathway, and cell survival and proliferation including Tibetans, Tibetan wild boars, ground tit, plateau fish and Yaks et al. [16–21]. However, little is known about the genetic and molecular mechanisms of hypoxia adaptation during embryonic brain development in indigenous animals, especially TBCs that adapt to high-altitude environments on the Qinghai-Tibet Plateau.

Although TBCs have adapted well to high altitudes for a long time, the genetic basis of adaptation to hypoxia remains unclear. To better understand the developmental pattern of TBC’s embryonic brain adaptation to hypoxia, TBCs and Dwarf Laying Chickens (DLCs) fertilized eggs were collected and hatched under normoxia (21%O_2_) and simulated high-altitude hypoxic environment (13%O_2_), respectively. Whole brain tissues at three stages of embryonic development (on days 8, 12, and 18 of development) were collected for transcriptome and metabolome analysis. This study aimed to explore the gene expression patterns and differences in the embryonic brain between TBCs and DLCs under different oxygen concentrations and developmental stages and further reveal the potential molecular mechanism of TBCs adapting to hypoxia.

## Results

### Illumina sequencing and genome-guided assembly

A total of 1,866,336,882 clean reads (279.75 Gb of data bulk) with a length of 250 ~ 300 bp were generated from the 36 libraries divided into 12 groups, and approximately 90% of the clean reads were mapped to the reference genome (Table S1). Gene expression levels were quantified using fragments per kilobase of exon per million mapped reads (FPKM). Besides, to evaluate the reliability of RNA-seq results, eight genes were selected randomly and qRT-PCR was performed using aliquots of non-pooled RNA samples (n = 6 for each group). Expression patterns were consistent with expression levels calculated from RNA-seq data, which indicated that RNA-seq could provide reliable data for mRNA differential expression analysis (Figure S1 and Table S2).

### Differential expression analysis of genes in the embryonic brain between TBCs and DLCs

**Fig. 1:**
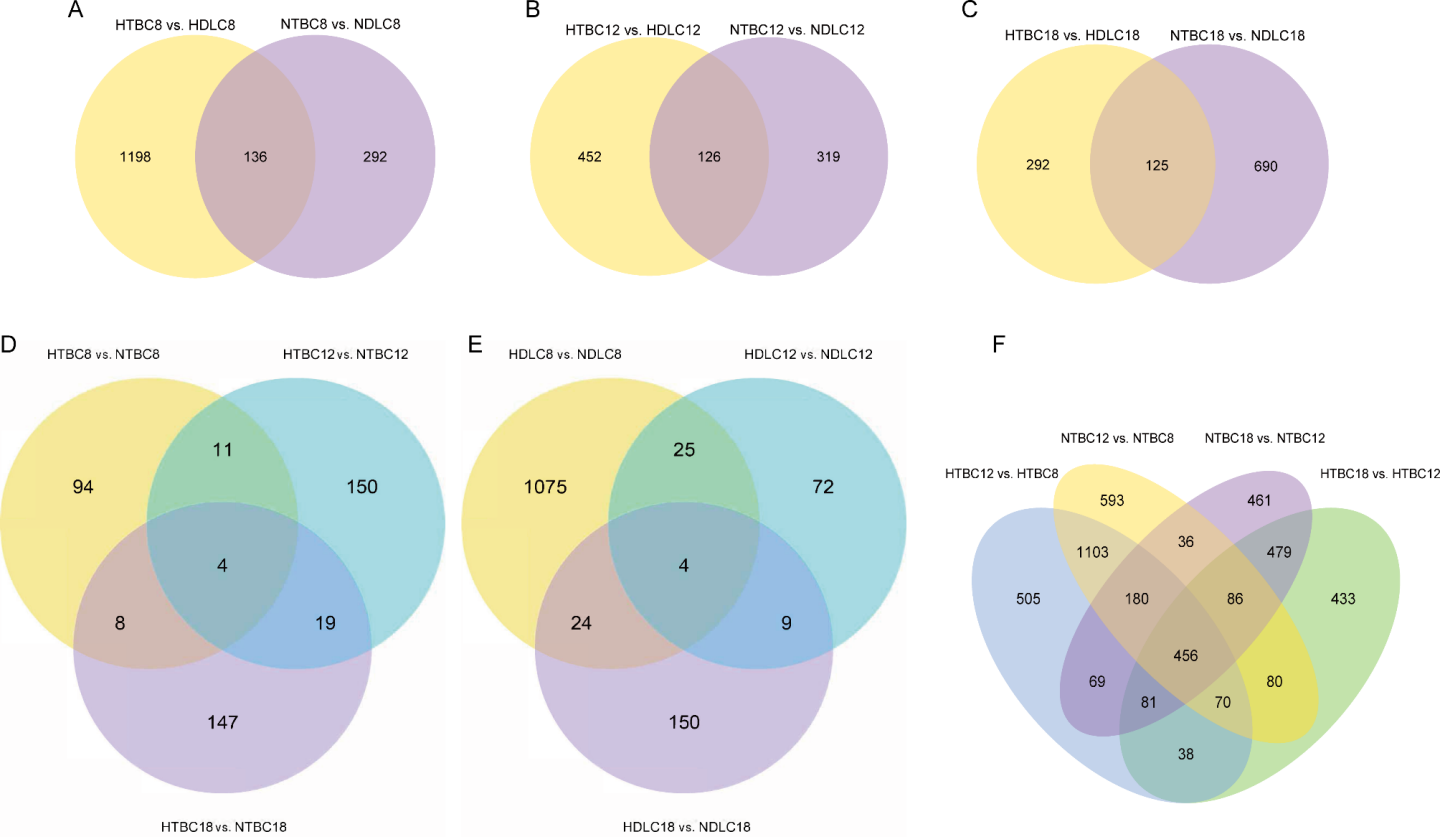
Identification of DEGs in TBCs and DLCs. (**A**-**C**) the DEGs between normoxia and hypoxia on days 8,12,18 of incubation respectively after TBCs compared to DLCs; (**D**-**E**) the DEGs between normoxia and hypoxia in the incubation of day 12 compared to day 8 and day 18 compared to day 12 in TBCs; (**F**) the DEGs on days 8,12,18 of incubation respectively in TBCs and DLCs after hypoxia compared to normoxia. HTBC and NTBC: Tibetan chicken in hypoxia and normoxia; HDLC and NDLC: Dwarf Laying Chicken in hypoxia and normoxia

In this study, we first compared the differentially expression genes (DEGs) between TBCs and DLCs under normoxic and hypoxic development conditions (Fig. 1A-C and Table S3). A total of 36 samples divided into 12 groups including TBCs and DLCs on day 8 (NTBC8 and NDLC8), day 12 (NTBC12 and NDLC12), and day 18 (NTBC18 and NDLC18) of development under normoxia and TBCs and DLCs on day 8 (HTBC8 and HDLC8), day 12 (HTBC12 and HDLC12), and day 18 (HTBC18 and HDLC18) of development under hypoxia, respectively. In normoxia, there were 428 DEGs in NTBC8 compared to NDLC8 (313 upregulated and 115 downregulated), 445 DEGs in NTBC12 compared to NDLC12 (256 upregulated and 189 downregulated), and 815 DEGs in NTBC18 compared to NDLC18 (464 upregulated and 351 downregulated). In hypoxia, there were 1334 DEGs in HTBC8 compared to HDLC8 (425 upregulated and 909 downregulated), 578 DEGs in HTBC12 compared to HDLC12 (295 upregulated and 283 downregulated), and 417 DEGs in HTBC18 compared to HDLC18 (248 upregulated and 169 downregulated). Furthermore, there were 1198 (324 upregulated, 874 downregulated ), 452 (232 upregulated, 220 downregulated ), and 292 (180 upregulated, 112 downregulated ) DEGs unique to hypoxia in each of the three periods between TBCs and DLCs compared with normoxia.

We then compared the DEGs between normoxia and hypoxia in TBCs and DLCs (Fig. 1D-E and Table S4). There were 117 (56 upregulated, 61 downregulated), 184 (78 upregulated, 86 downregulated), and 178 (98 upregulated, 80 downregulated) DEGs in HTBC8 compared to NTBC8, HTBC12 compared to NTBC12, and HTBC18 compared to NTBC18, respectively (Fig. 1D), and 1128 (1015 upregulated, 113 downregulated), 110 (60 upregulated, 50 downregulated), and 187 (119 upregulated, 68 downregulated) DEGs in HDLC8 compared to NDLC8, HDLC12 compared to NDLC12, and HDLC18 compared to NDLC18, respectively (Fig. 1E).

Finally, we compared the DEGs at different development stages in the TBCs (Fig. 1F and Table S5). There were 2604 (1847 upregulated ,757 downregulated ) and 1848 (1223 upregulated, 625 downregulated ) DEGs in NTBC12 compared to NTBC8 and NTBC18 compared to NTBC12 in normoxia, respectively, and 2502 (1752 upregulated ,750 downregulated ) and 1723 (1187 upregulated, 536 downregulated ) DEGs in HTBC12 compared to HTBC8 and HTBC18 compared to HTBC12 in hypoxia, respectively. In addition, there were 693 (375 upregulated, 318 downregulated ) and 621 (377 upregulated, 244 downregulated ) DEGs unique in HTBC12 compared to HTBC8 and HTBC18 compared to HTBC12 in TBCs under hypoxia after comparison with normoxia, respectively.

### Functional analysis of DEGs

#### Functional analysis of DEGs between TBCs and DLCs

To further understand the biological functions of DEGs, we first performed DEGs analysis between TBCs and DLCs in the development incubation stage through GO enrichment and KEGG pathway analysis (Figure S2 and Table S6 and S9). In normoxia, 68 significant GO terms (p < 0.05) were detected in NTBC8 compared with NDLC8, with the most significant enrichment focusing on cell adhesion (Figure S2A). The enriched KEGG pathways included cytokine-cytokine receptor interactions and neuroactive ligand-receptor interactions (Figure S2D). When comparing NTBC12 and NDLC12, 192 significant GO terms were mainly involved in the side of the membrane, cell surface, and immune response (Figure S2B). KEGG pathways containing phagosomes and ECM-receptor interactions were enriched (Figure S2E). There were 237 significant GO terms mainly involved in the ECM, organic acid transport, and sensory perception, and three enriched pathways involved in the phagosome, cytokine-cytokine receptor interaction, and fatty acid elongation compared to NTBC18 and NDLC18 (Figure S2C and F).

**Fig. 2:**
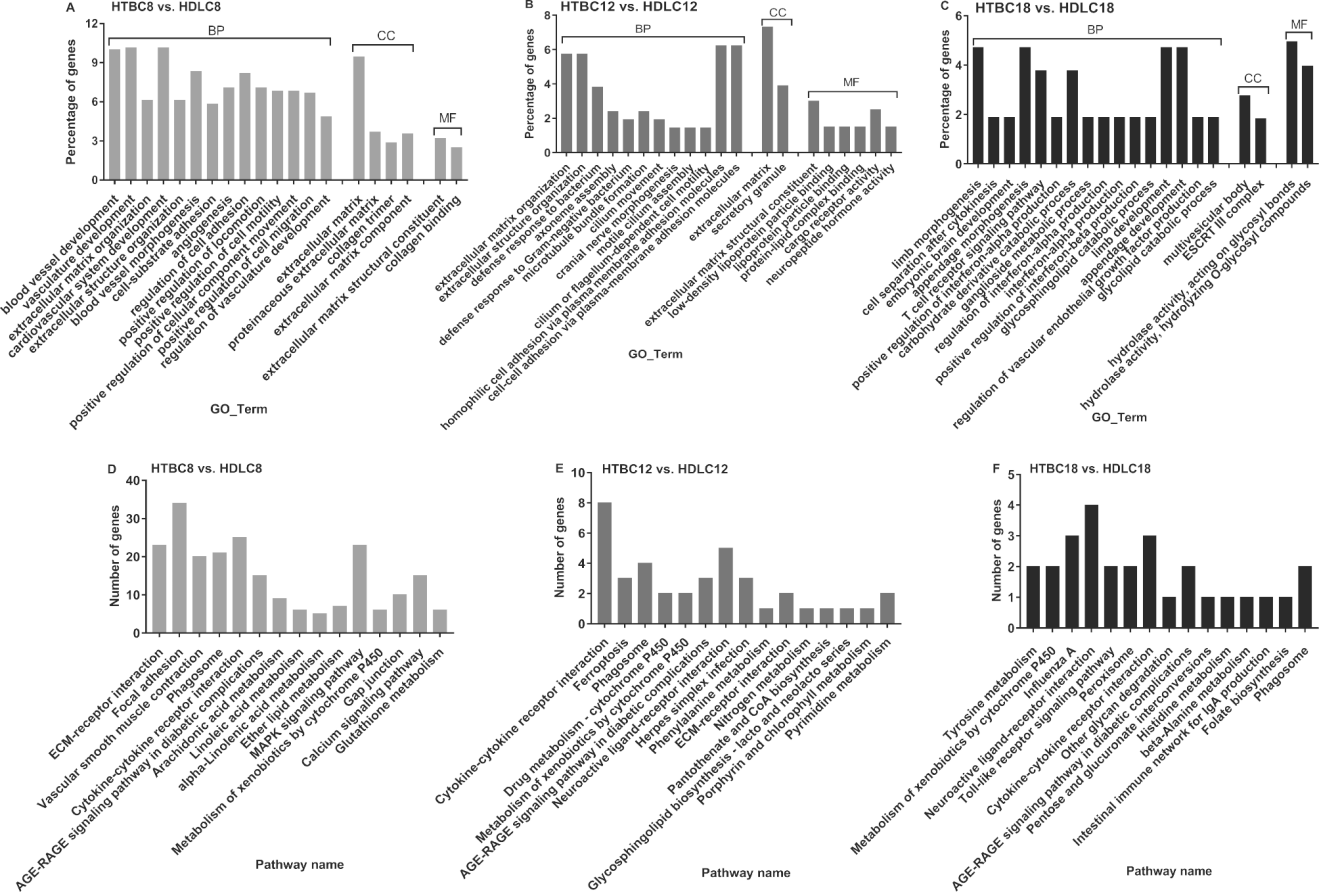
The top 20 of classification of gene ontology (GO) in three main categories and the top 15 pathways of DEGs on days 8, 12, and 18 of incubation between (**A** and **D**) HTBC8 and HDLC8, (**B** and **E**) HTBC12 and HDLC12, and (**C** and **F**) HTBC18 and HDLC18. The complete list of GO assignments can be found in the Supplementary Materials. (BP: biological process, CC: cellular component, and MF: molecular function)

In hypoxia (Fig. 2 and Table S6 and S9), comparing HTBC8 and HDLC8, GO terms of unique 1198 DEGs were significantly enriched and focused on vessel development, cell migration, and ECM (Fig. 2A). There were enriched KEGG pathways involved in focal adhesion, vascular smooth muscle contraction, glycolysis/gluconeogenesis, glutathione metabolism, and the MAPK signaling pathway (Fig. 2D). Comparing HTBC12 and HDLC12, 104 GO terms of unique 452 DEGs were significantly enriched, focusing on defense response to bacteria, oxygen transport and binding, and ECM and cell adhesion (Fig. 2B). The enriched pathways were mainly involved in cytokine-cytokine receptor interactions and cytochrome P450 and Neuroactive ligand-receptor interactions (Fig. 2E). Comparing HTBC18 and HDLC18, the significant GO terms of unique 292 DEGs mainly focused on hydrolase activity, embryonic brain development, T cell receptor signaling pathway, appendage morphogenesis, and interferon-alpha production (Fig. 2C). Tyrosine metabolism was the most significantly enriched pathway (Fig. 2F).

### Functional analysis of DEGs between normoxia and hypoxia

**Fig. 3:**
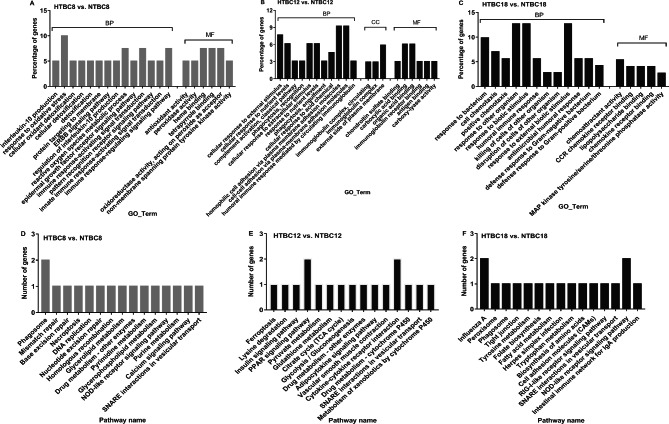
The top 20 of classification of gene ontology (GO) in three main categories and the top 15 pathways of DEGs on days 8, 12, and 18 of incubation between (**A** and **D**) HTBC8 and NTBC8, (**B** and **E**) HTBC12 and NTBC12, and (**C** and **F**) HTBC18 and NTBC18. The complete list of GO assignments can be found in the Supplementary Materials. (BP: biological process, CC: cellular component, and MF: molecular function)

We also performed a DEGs analysis of the embryonic brain between normoxia and hypoxia in TBCs and DLCs (Table S7 and S10). In TBCs, 79 GO terms were significantly enriched, mainly focusing on oxidoreductase and antioxidant activity, oxidative stress, and oxygen transport, compared to HTBC8 and NTBC8 (Fig. 3A). The most significantly enriched pathway was phagosomes (Fig. 3D). Comparing HTBC12 and NTBC12, 51 GO terms were significantly enriched, focusing on the cellular response to external stimuli, response to nutrient levels, and cell adhesion (Fig. 3B). The PPAR signaling pathway, a classic signaling pathway, was also significantly enriched (Fig. 3E). A total of 41 GO terms were significantly enriched, mainly related to the defense response to bacteria, lipopolysaccharide binding, and MAP kinase phosphatase activity, and the most significantly enriched pathway was the NOD-like receptor signaling pathway compared to HTBC18 and NTBC18 (Fig. 3C and F).

In DLCs (Tables S7 and S10), GO terms were significantly enriched, focusing on blood vessel development, growth factor binding, embryonic organ development, response to oxygen levels, ECM, and KEGG pathways, including vascular smooth muscle contraction, MAPK signaling pathway, glycolysis/gluconeogenesis, and glutathione metabolism were also significantly different between HDLC8 and NDLC8. Compared to HDLC12 and NDLC12, GO terms were significantly enriched, focusing on defense responses to bacteria, lipopolysaccharide binding, oxygen transport, and biotic stimuli. The KEGG pathways were enriched for tyrosine metabolism. Comparing HDLC18 and NDLC18, GO terms were significant enrichment focuses on G-protein coupled receptor binding, neuropeptide receptor binding, glucose process, glutathione transferase activity, the pathway related to Glutathione metabolism, and the cytochrome P450 were significantly enriched.

### Functional analysis of DEGs between three incubation stages in TBCs

We finally performed the DEGs analysis of the TBCs brain between the three stages of incubation during embryo development through GO enrichment and KEGG pathway analysis (Table S8 and S11). In normoxia, compared with NTBC8 and NTBC12, significant enrichment focuses on synaptic processes, vascular development, and nervous system development. Comparing NTBC12 and NTBC18, significant enrichment in addition to synaptic processes and vascular development, there were also GO terms detected for brain development, GABA, and GABA-A receptor, which regulate brain function and development, and the neuroactive ligand-receptor interaction and calcium signaling pathway were also significantly enriched.

**Fig. 4:**
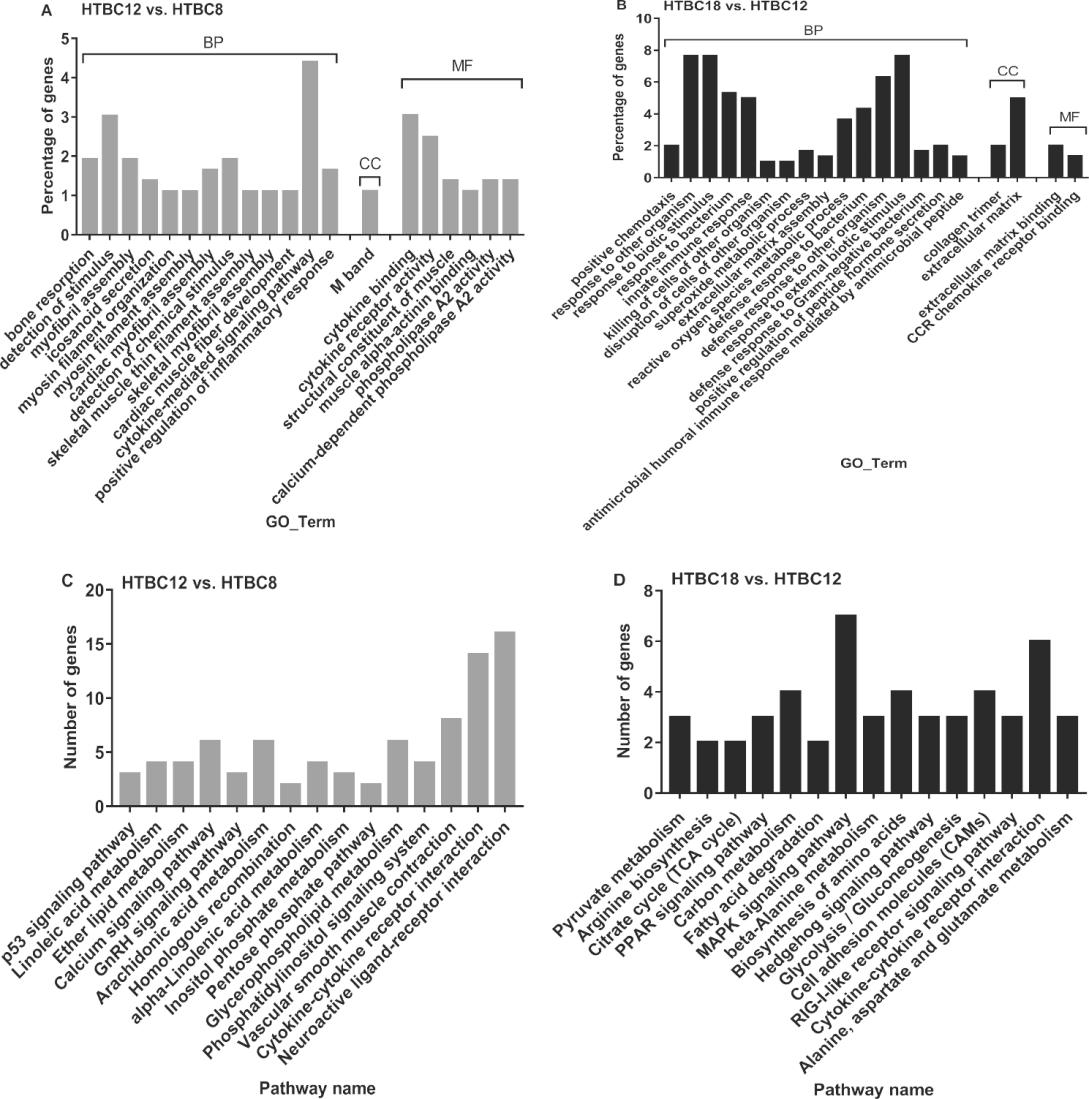
The top 20 of classification of gene ontology (GO) in three main categories and the top 15 pathways of DEGs on days 8, 12, and 18 of incubation between (**A** and **C**) HTBC12 and HTBC8, (**B** and **D**) HTBC18 and NTBC12. The complete list of GO assignments can be found in the Supplementary Materials. (BP: biological process, CC: cellular component, and MF: molecular function)

In hypoxia, compared to HTBC8 and HTBC12, significantly enriched GO terms of 693 unique DEGs were detected for calcium ion binding and cytokine-mediated signaling pathways, and there were significantly enriched KEGG pathways involved in linoleic acid, arachidonic acid, and glycerophospholipid metabolism (Fig. 4A and C). Comparing HTBC18 and HTBC12, in addition to biotic stimulus, response to the bacterium, and ECM, 632 unique DEGs were significantly enriched in the superoxide metabolic process and significant KEGG pathways involved in pyruvate metabolism were detected (Fig. 4B and D).

### Regulatory network of DEGs analysis

**Fig. 5:**
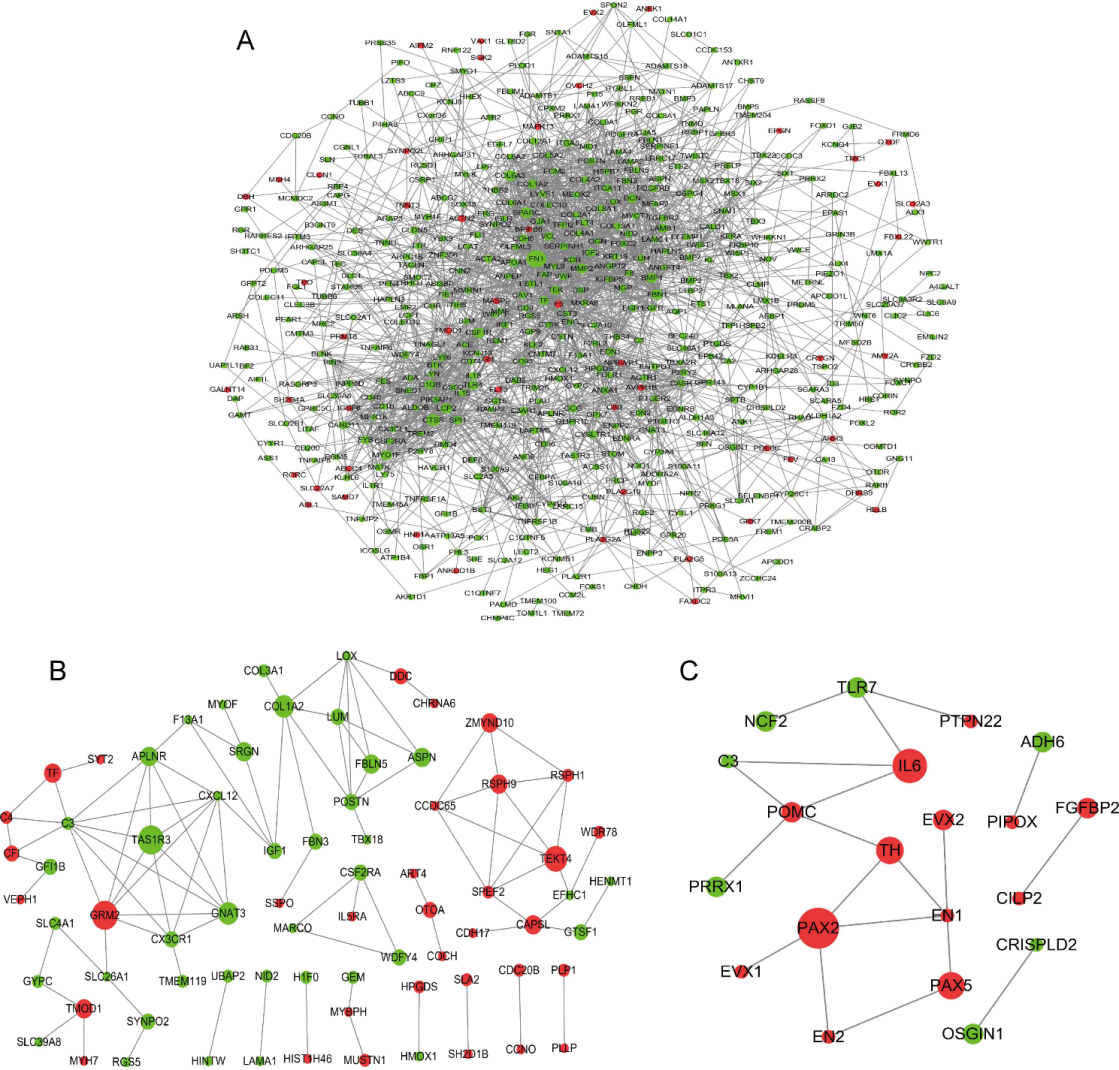
Regulatory network of DEGs in the three incubation periods of days 8, 12, and 18 between TBCs and DLCs under hypoxia. (**A**) Interaction network of DEGs between HTBC8 and HDLC8; (**B**) Interaction network of DEGs between HTBC12 and HDLC12; (**C**) Interaction network of DEGs between HTBC18 and HDLC18. Nodes (circles) represent the proteins encoded by DEGs. The radius of the circle indicates the significance of enrichment, red indicates that the expression of DEGs is relatively more abundant, and green indicates that the expression of DEGs is relatively less abundant in TBCs than in DLCs. DEG, differentially expressed gene

The significant DEGs were used to construct a protein-protein interaction (PPI) network. Dozens of gene nodes showed high degrees of connectivity in hypoxia between TBCs and DLCs (Fig. 5), including *FN1*, *CTSS*, *LCP2*, *C1QB*, *FYB*, *FBN1*, *COL6A3*, *BMP4*, *F5*, *TNNT3* on day 8 (Fig. 5A), *TAS1R3*, *GNAT3*, *COL1A2*, *FBLN5*, *GRM2*, *TEKT4*, *TMOD1*, *CAPSL* on day 12 (Fig. 5B), and *ADH6*, *TLR7*, *PRRX1*, *PAX2*, *IL6*, *PAX5*, *POMC* on day 18 (Fig. 5C).

**Fig. 6:**
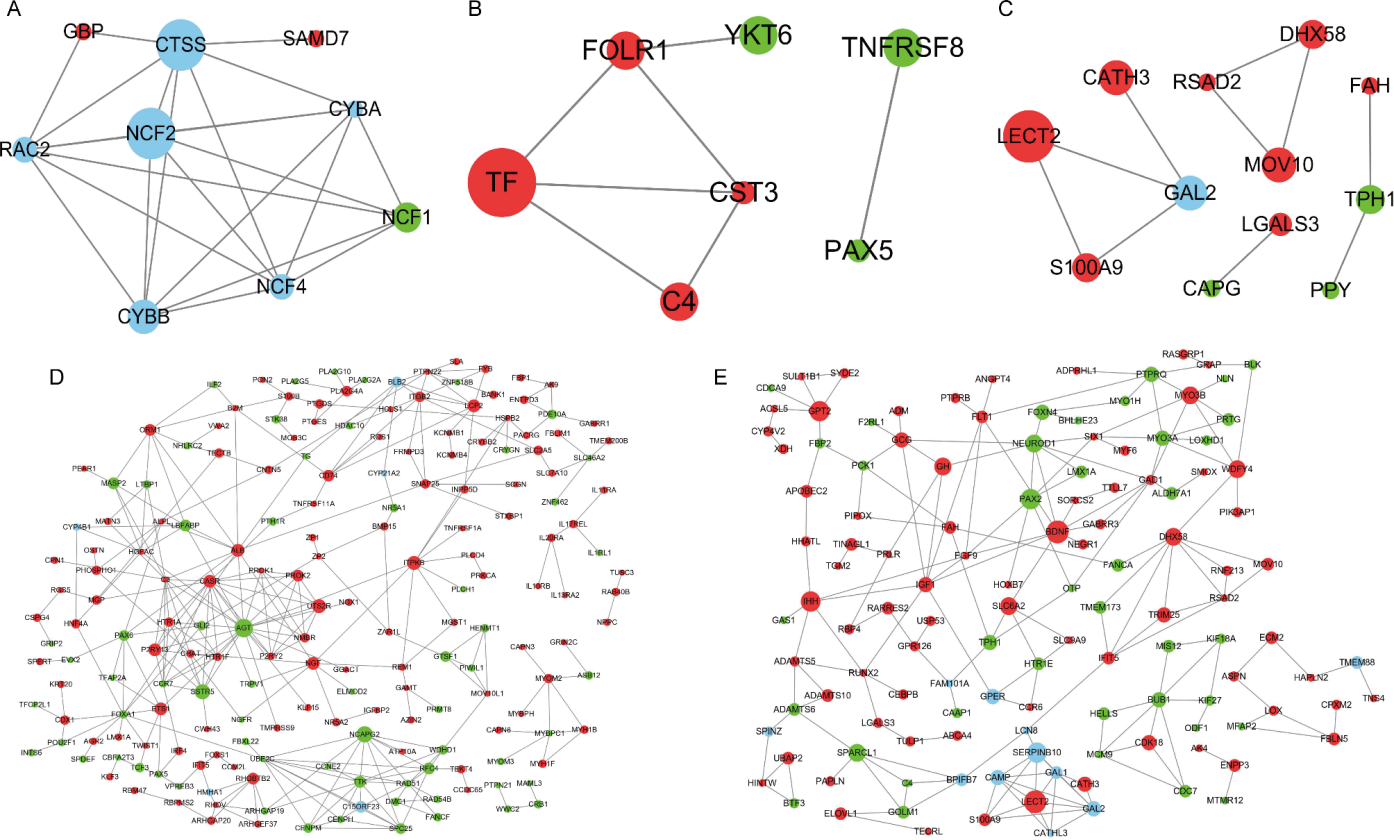
Regulatory network of DEGs in the three incubation periods of days 8, 12, and 18 in TBCs under hypoxia and normoxia. (**A**) Interaction network of DEGs between HTBC8 and NTBC8; (**B**) Interaction network of DEGs between HTBC12 and NTBC12; (**C**) Interaction network of DEGs between HTBC18 and NTBLC18; (**D**) Interaction network of DEGs between HTBC12 and HTBC8; (**E**) Interaction network of DEGs between HTBC18 and HTBLC12. Nodes (circles) represent the proteins encoded by DEGs. The radius of the circle indicates the significance of enrichment, red indicates that the expression of DEGs is relatively more abundant, green indicates that the expression of DEGs is relatively less abundant, and blue indicates other. DEG, differentially expressed gene

We also analyzed the DEGs between hypoxia and normoxia in TBCs and DLCs and found some genes with high connectivity degrees (Fig. 6), including *NCF1*, *GBP*, *SAMD7* between HTBC8 and NTBC8 (Fig. 6A); *TNFRSR8*, *YKT6*, *TF*, *FOLR1*, *C4* between HTBC12 and NTBC12 (Fig. 6B), *TPH1*, *LECT2*, *CATH3*, *MOV10*, *DHX58* between HTBC18 and NTBC18 (Fig. 6C); *FN1*, *CTSS*, *LCP2*, *C1QA*, *TLR4*, *CSF1R*, *FBN1*, *COL6A3*, *ADIPOQ*, *MYO1F*, *AGT* between HDLC8 and NDLC8 (Figure S3A), *LECT2*, *CATH3*, *DBH* between HDLC12 and NDLC12 (Figure S3B); and *GCG*, *EGR1*, *HCRT*, *NOX1*, *POMC* between HDLC18 and NDLC18 (Figure S3C). Finally, significant DEGs between the three stages of incubation in TBCs under hypoxic conditions were used to construct the PPI network (Fig. 6). The gene nodes had high degrees of connectivity, including *AGT*, *SSTR5*, *NCAPG2*, *ETS1*, *CASR*, *ALB*, *UST2R*, *ITPKB* between HTBC12 and HTBC8 (Fig. 6D) and *PAX2*, *NEUROD1*, *MYO3A*, *SPARCL1*, *GPT2*, *IHH*, *BDNF*, *DHX58*, *MYO3B* between HTBC18 and HTBC12 (Fig. 6E).

### TBCs have higher energy metabolism than DLCs during hypoxia

Observing DEGs enrichment to metabolism and oxygen transport pathways between TBCs and DLCs under hypoxia, metabolome was used to examine glycolysis and the Krebs cycle which are important reaction processes related to energy metabolism and oxygen consumption. The metabolome data first published in our previous article [22] indicated that compared with DLCs under hypoxia, TBCs had significantly high metabolite concentrations including pyruvic acid, lactic acid, citric acid, α-ketoglutaric acid and succinic acid on day 8, glucose-6-phosphate(G6P), fructose-6-phosphate(F6P), pyruvic acid, lactic acid, isocitric acid, succinic acid, fumaric acid and malic acid on day12, lactic acid, citric acid and succinic acid on day18. Surprisingly, DLCs had significantly high concentration of G6P acid on day18. Under normoxia, the result was the opposite of hypoxia. Especially on day 12, DLCs had significantly high concentrations of G6P, F6P and lactic acid. Together, compared to DLCs, above results suggested that TBCs had higher energy metabolism than DLCs during hypoxia.

## Discussion

As the altitude increases, the oxygen concentration gradually decreases, and the amount of oxygen available to the animal decreases, resulting in hypoxic stress, which causes various dysfunctions, affecting normal growth, development, and even death [23]. Indigenous animals, such as TBCs, live on the Qinghai-Tibet Plateau and adapt to a low-oxygen environment by adjusting their physiology [2, 24, 25]. In this study, we investigated the development patterns of embryonic brain at different stages by integrating analysis of the transcriptome with metabolome data. Among NTBC8 and NDLC8, the most diverse DEGs were involved in cell adhesion, such as *PTPN6, CD80, GCNT1*, etc. Cell adhesion is the molecular basis of tissue architecture and morphogenesis, which is crucial for the assembly of individual cells into three-dimensional tissues of animals [26, 27]. A proportion of key genes were significantly involved in the side of the membrane, cell surface, and ECM, for example, *BG1, BTN1A1*, and *COLEC12*, but also related to immune response compared to NTBC12 with NDLC12. As the noncellular component is present in all tissues and organs, the ECM provides essential physical scaffolding for cellular constituents [28, 29]. Between NTBC18 and NDLC18, the most DEGs involved in ECM for *SCARA3, COL1A2, COL3A1* etc. Collagen (COL) is the main structural and load-bearing element of various connective tissues, where it forms the ECM that supports cells [30]. The above results indicate that the differences were mainly in non-cellular components between these two chicken breeds during embryonic brain development under normoxia. This may be because TBCs live on the plateau for many generations, and the DLCs grow in the plain for a long time, which formed a genetic background difference.

Due to the genetic background differences between the two chicken breeds, we analyzed and compared the unique DEGs of TBCs and DLCs after hypoxic stimulation. There were differences in the gene expression profiles of hypoxia at the same stage of development between TBCs and DLCs. Most vital genes, such as *FBP1, EPAS1, VEGFD, ALDH1A2, GPX7, PCK1, ALDOB*, etc., are significantly involved in glycolysis/gluconeogenesis, glutathione metabolism, calcium signaling pathways, vasculature development for HTBC8 and HDLC8 (Table 1). Metabolomics data also showed the same results, with differences in energy metabolism includ-ing glycolysis and Krebs cycle between HTBC8 and HDLC8, which was consistent with our previous work [22, 31]. In this vital genes, *FBP1* is a rate-limiting enzyme in gluconeogenesis and is an important process in cell energy metabolism. *FBP1* can act as an HIF repressor in the nucleus by binding to the HIF inhibitory domain to adapt to hypoxic environments [32]. *EPAS1* encodes HIF-2a, which is involved in the hypoxia-inducible factor (HIF) pathway and shows the strongest signals of selective sweeps in Tibetans [33, 34]. Some studies have shown that *EPAS1* is associated with the genetic adaptation of animals to high altitudes [18, 35, 36]. *VEGFD* is one of five additional members of the vascular endothelial growth factor (*VEGF*) family, and *VEGF* is a member of the platelet-derived growth factor family and is a major inducer of angiogenesis and vessel permeability [37]. HIF-1α functions via the regulation of *VEGFD* as a cellular homeostatic effector in response to hypoxia [38].

**Table 1:** Significantly enriched GO terms and KEGG pathways of DEGs in HTBC8 vs. HDCL8

Description	Terms	Genes
vessel development	GO:0001568	*COL1A2, MMP2, DCN, COL4A1, SPARC, ANGPT2, CDH5, EDNRA, ACTA2, GJA1, VEGFD, CYP1B1, ALDH1A2, MYLK, PDGFRA, APLNR, ADGRA2, EMP2, THBS4, TEK, TIE1*, *EPAS1, LAMA4, ELK3, AQP1, KDR, PRRX1, SIX1, BMP4, SMOC2, TMEM100, TNMD, ETS2, OSR1, ANGPT4, PRCP, ENPP2, JCAD, FLT4*
	GO:0001944	
	GO:0048514	
Vascular smooth muscle contraction	gga04270	*CALD1, EDNRA, ACTA2, MYL9, MYLK, ITPR3, RAMP2, PRKG1, MRVI1, ADORA2A*, *NPR2, PLA2G5, PLA2G10, PLA2G2A, AGTR1,KCNMB1, AVPR1B*
MAPK signaling pathway	gga04010	*ANGPT2, VEGFD, PDGFRA, PDGFRB*, *EGFR, TEK, CSF1R, KDR, NRK, ANGPT4*, *FLT4, IGF2, TGFBR2, RASGRP3, TNFRSF1A*, *IL1R1, IGF-I, ARR3, MAPK13, FLT3*
Calcium signaling pathway	gga04020	*EDNRA, MYLK, PDGFRA, PDGFRB, EGFR*, *ITPR3, EDNRB, ADORA2A, PTGER3, TBXA2R, OXTR, CYSLTR1, AGTR1, AVPR1B*
Glutathione metabolism	gga00480	*GGT5, ANPEP, GSTA4L*, *GSTA3, GPX7, HPGDS*
Glycolysis / Gluconeogenesis	gga00010	*FBP1, ACSS1, ALDOB, ALDH1A3, PCK1*

Comparing HTBC12 and HDLC12 (Table 2), oxygen transport and binding for *HBZ, HBBR*, glucose homeostasis for *NDUFAF2, PIK3CA, IDH1, ALDOC*, the defense responses to bacteria for *AvBD1, CATH3, COCH, etc.*, and cytokine-cytokine receptor interactions for *IL1R1, IL5RA, CXCL12* were significantly enriched in the GO terms. *NDUFAF2* is a chaperone involved in the assembly of NADH dehydrogenase (ubiquinone) also known as complex I [39], IDH1 is an enzyme that catalyzes the oxidative decarboxylation of isocitrate to 2-oxoglutarate in Krebs cycle [40]. This suggested that there may be differences in mitochondrial metabolism between TBCs and DLCs under hypoxia. Our previous work has also demonstrated TBCs had higher TCA cycle activities and mitochondrial quality compared to DLCs during hypoxia [22], which is consistent with this. *AvBD1, CATHL2*, and *CATH3* associated with antibacterial activity were upregulated in DLCs, implying that hypoxia was a more irritating stimulus for DLCs that required more reactions to adapt to it, and it also showed that TBCs had stronger hypoxia tolerance. There were also DEGs related to ECM for *FBLN5, COL1A2, COL3A1, POSTN*, and *FBN3*. Fibulin-5 (*FBLN5*), which is significantly expressed under hypoxia and is also detected on day 8, is an ECM protein essential for elastic fiber assembly and vasculogenesis that participates in vascular remodeling and controls endothelial cell adhesion, motility, and proliferation and *FBLN5* induction could be involved in the adaptive survival response of endothelial cells to hypoxia [41].

**Table 2:** Significantly enriched GO terms and KEGG pathways of DEGs in HTBC12 vs. HDCL12

Description	Terms	Genes
response to bacterium	GO:0042742	*CATHL1, AvBD1, AvBD2, CATHL2*, *BD7, CATH3, RNASE4, COCH*
	GO:0009617	*CATHL1, AvBD1, AvBD2, CATHL2*, *BD7, CATH3, RNASE4, COCH, LY86*, *C3, CX3CR1*
extracellular matrix	GO:0031012	*NID2, ABI3BP, LRRC17, ECM2, LUM, COL1A2, CRISPLD2, COCH, FBLN1, POSTN, COL6A1, LOX, ASPN, MARCO, WNT6, FBLN5*
oxygen transport and binding	GO:0015669	*ENSGALG00000035309, HBZ, HBBR*
	GO:0019825	
Cytokine-cytokine receptor interaction	gga04060	*TNFRSF1B, CXCL12, IL1R1, TNFRSF8*, *IL5RA, IL1RL1, CSF2RA, CX3CR1*
Drug metabolism - cytochrome P450	gga00982	*HPGDS, FMO3, ENSGALG00000028858*
Neuroactive ligand-receptor interaction	gga04080	*GRM2, OXTR, APLNR, CHRNA6, PGR2, ENSGALG00000019797*
glucose homeostasis	gga00480 gga00010	*NDUFAF2, PIK3CA, IDH1, ALDOC*

*IL6, TLR7, IL5RA*, and *EN1* are significantly involved in the immune response and brain development compared to HTBC18 and HDLC18 (Table 3). *IL-6* is a pleiotropic cytokine produced by immune and blood cells, endothelial cells, and myocytes during contraction, and can cross the blood-brain barrier with various biological activities in immune regulation, hematopoiesis, and inflammation, as well as metabolic, proliferative, and regenerative processes [42]. Hypoxia induces *IL-6* release [43, 44]. *TLR7* is crucial in host defense against viruses, whose downstream signaling leads to dramatic cellular stress associated with energy consumption [45]. *TLR7* downstream signaling can induce the expression and accumulation of transcriptionally active HIF-1α to mediate the inflammatory response [46]. Glycolysis / Gluconeogenesis for *MINPP1, ADH6, LDHA, FBP1* were also enriched. *LDHA* has been proven to be the targets of HIF-1 in mammals and our ChIP assay results indicated that HIF-1 could also bind to *HK1* and *LDHA* in chickens [31].

**Table 3:** Significantly enriched GO terms and KEGG pathways of DEGs in HTBC18 vs. HDCL18

Description	Terms	Genes
embryonic morphogenesis	GO:0035108	*EN1, EVX2, PRRX1, CRABP2*, *PITX1, OSR1, PITX2*
	GO:0030326	
	GO:0060173	
T cell receptor signaling pathway	GO:0050852	*ENSGALG00000028157*, *ENSGALG00000039480*, *ENSGALG00000024365, SKAP1*, *ENSGALG00000013101, PTPN22*
hydrolase activity	GO:0016798	*ENSGALG00000036536*, *ENSGALG00000025921, GM2A,NEU4*,
Cytokine-cytokine receptor interaction	gga04060	*novel.26, IL5RA, PRLR*, *novel.657, IL6*
Tyrosine metabolism	gga00350	*TH, ADH6*
Glycolysis / Gluconeogenesis	gga00010	*MINPP1, ADH6, LDHA, FBP1*

The most significant difference in oxidative stress for *EPX, TPO, INAVA*, and *HBE1* between HTBC8 and NTBC8, yet it was a notable change in vascular system development for *EPAS1, VEGFD, MMP2, PDGFRA, ANGPT2, ANGPT4, EDN1* between HDLC8 and NDLBC8. Hypoxia may seriously affect the vascular development of DLCs, making them unable to deliver enough oxygen to supply brain development, which may be the reason for the higher mortality of DLCs than TBCs. This result is consistent with those of our previous studies [22, 31]. In the comparison of HTBC12 with NTBC12, most vital genes such as *PCK1, FOLR1, FABP6*, and *TNFRSF8* were involved in nutrient levels. Moreover, comparing HDLC12 with NDLC12, the DEGs were also mainly related to the response to bacteria for *CATHL1, CATH3, AvBD1, COCH*, and *CD14*. Among HTBC18 and NTBC18, most DEGs were also mainly involved in the response to bacteria for *AvBD1, AvBD2, CATHL1 etc.* indicating that carbohydrate homeostasis, including glucose, was mutated for *GCG, PRCP, POMC, HNF4A* compared to HDLC18 with NDLC18. Glucose is the primary source of energy for brain tissue [47, 48] and hypoxia may block the glucose metabolism of DLCs, making it unable to produce enough energy to supply brain development, which may be the reason for the higher mortality rate of DLCs than TBCs on day 18. These results indicated that the expression patterns of TBCs and DLCs were different during the same period of hatching compared to hypoxia and normoxia, respectively.

In both normoxic and hypoxic incubation, the embryonic brain changed the most in synaptic signaling as the incubation time increased in TBCs. However, the results changed in the three stages of incubation under hypoxia after comparison with normoxia. Pathway analysis showed that fatty acid metabolism pathways (*PLA2G2A, PLA2G4A, PLA2G5*, and *PLA2G10*) were significantly associated with DEGs between HTBC12 and HTBC8, while amino acid metabolism and synthesis (*GAD1, SMOX, PCK1, GPT2*, and *ALDH7A1*) were significantly associated with HTBC18 and HTBC12. Phospholipase A2 (*PLA2*) converts phospholipids to generate free fatty acids and lysophospholipids and plays a fundamental role in cell injury in the central nervous system. Activation of *PLA2* during hypoxia plays an important role in the processes responsible for generating free fatty acids [49]. These results indicate that under hypoxia, TBCs have different gene expression patterns at different stages of brain development.

## Conclusion

Through transcriptome and metabolome analysis, we focused on hypoxia adaptation profiling of the embryonic brain under hypoxia at three incubation time points in TBCs and DLCs. In summary, our results showed that under development incubation, TBCs and DLCs had different gene expression patterns during the same period, with differences mainly in metabolism (glycolysis, glutathione metabolism, etc.), vascular development, antibacterial response, brain and brain neurodevelopment, and immune response. We found some key DEGs between TBCs and DLCs, such as *EPAS1, VEGFD, FBP1, FBLN5, LDHA* and *IL-6* which are involved in the HIF pathway and hypoxia regulation. Moreover, compared to DLCs, the development of TBCs under hypoxia were less variable than those under normoxia, suggesting that TBCs are more adaptable to hypoxia and that DLCs require greater efforts to respond to hypoxia. These results provide a basis for uncovering the molecular regulation mechanism of hypoxia adaptation in TBCs, and a potential application of hypoxia adaptation research for other animals living on the Qinghai-Tibet Plateau, and may even contribute to the study of brain diseases caused by hypoxia.

## Materials and methods

### Animals and sample collection

Embryonic whole brain tissue at three stages of embryonic development (on days 8, 12, and 18 of development) are collected for next research as previously described [31]. Fertilized eggs from TBCs and DLCs were collected at the Experimental Chicken Farm at China Agricultural University (CAU) and were transferred to normoxia (21% O_2_) and hypoxia (13% O_2_) incubators. 100 eggs were incubated from each breed per condition. Temperature was maintained at 37.8 °C with a relative humidity of 60%.

### Sample preparation for GC-MS and metabolomics analysis

Sample preparation and GC-MS analysis was described in the previous published work [22].

### RNA extraction

Total RNA was extracted from each sample using the TRIzol reagent (Invitrogen, CA, USA) and RNA degradation and contamination was monitored on 1% agarose gels. RNA purity was checked using the NanoPhotometer® spectrophotometer (IMPLEN, CA, USA). RNA concentration was measured using Qubit® RNA Assay Kit in Qubit®2.0 Flurometer (Life Technologies, CA, USA) and the integrity was assessed using the RNA Nano 6000 Assay Kit of the Bioanalyzer 2100 system (Agilent Technologies, CA, USA).

### Library preparation and transcriptome sequencing

A total amount of 3 µg RNA per sample was used as input material for the RNA sample preparations. Sequencing libraries were generated using NEBNext® UltraTM RNA Library Prep Kit (NEB, Ipswich, MA, USA) following manufacturer’s recommendations and according to previous article [50]. Briefly, mRNA was purified from total RNA using poly-T oligo-attached magnetic beads. RNA was fragmented to generate short RNA strands using NEBNext First Strand Synthesis Reaction Buffer (NEB, Ispawich, MA, USA). First strand cDNA was synthesized using random hexamer primer and M-MuLV Reverse Transcriptase (RNase H-) and second strand cDNA synthesis was subsequently performed using DNA Polymerase I and RNase H. Remaining overhangs were converted into blunt ends via exonuclease/polymerase activities. After adenylation of 3’ ends of DNA fragments, NEBNext Adaptor with hairpin loop structure were ligated to prepare for hybridization. In order to select cDNA fragments of preferentially 250 ~ 300 bp in length, the library fragments were purified with AMPure XP system (Beckman Coulter, Beverly, USA). Then 3 µl USER Enzyme (NEB, USA) was used with size-selected, adaptor-ligated cDNA at 37 °C for 15 min followed by 5 min at 95 °C before PCR. Then PCR was performed with Phusion High-Fidelity DNA polymerase, Universal PCR primers and Index (X) Primer. At last, PCR products were purified (AMPure XP system) and library quality was assessed on the Agilent Bioanalyzer 2100 system (Agilent Technologies).

### Sequence assembly and primary analysis

Raw data were firstly processed through in-house perl scripts by removing reads containing adapter, reads containing ploy-N and low quality reads using fastq (version 0.22.0). Q20, Q30 and GC content the clean data were calculated. All the downstream analyses were based on the clean data with high quality. Reference genome (ftp://ftp.ensembl.org/pub/release-108/fasta/gallus_gallus/dna/) and gene model annotation files (ftp://ftp.ensembl.org/pub/release-108/gtf/gallus_gallus/) were downloaded from genome website directly. Index of the reference genome was built and paired-end clean reads were aligned to the reference genome using Hisat2 v2.0.5 [51]. FeatureCounts v1.5.0-p3 was used to count the reads numbers mapped to each gene [52]. And then Fragments Per Kilobase of exon per Million fragments mapped (FPKM) of each gene was calculated based on the length of the gene and reads count mapped to this gene. Differential expression analysis was performed using the DESeq2 R package (1.16.1) and genes with a P-value < 0.05 and |log_2_ fold change| > 1 found by DESeq2 were assigned as differentially expressed [53].

### GO and pathway enrichment analysis of DEGs

GO enrichment analysis of DEGs was implemented by the clusterProfiler R package, in which gene length bias was corrected. GO terms with a P-Value less than 0.05 were considered significantly enriched by differential expressed genes. KEGG is a database resource for understanding high-level functions and utilities of the biological system, such as the cell, the organism and the ecosystem, from molecular-level information, especially large-scale molecular datasets generated by genome sequencing and other high-through put experimental technologies [54]. We used clusterProfiler R package (3.18.1) to test the statistical enrichment of differential expression genes in KEGG pathways.

### Regulatory network of DEGs analysis

PPI analysis of differentially expressed genes was performed using the STRING database (https://string-db.org), which contains known and predicted PPIs [55]. In our study, the STRING tool was used to perform PPIs among the DEGs and interactions with a combined score of ≥ 0.4. The network was constructed and visualized using Cytoscape v3.7.2 (https://cytoscape.org) [56].

### Validation of gene expression by qRT-PCR analysis

Gene primers (Table S12) for qRT-PCR were designed using Primer Premier 5.0 (Premier Biosoft International, Palo Alto, CA, USA) and were subsequently synthesized (Sangon Biotech, Beijing, China) for quantitative RT-PCR analysis. β-actin was used as an endogenous control for normalization and the cycling parameters used for qPCR amplification were as follows: initial heat denaturation at 95 °C for 15 min, 40 cycles at 95 °C for 30 s, 60 °C for 30 s, and 72 °C for 30 s; and a final extension at 72 °C for 5 min according to previous articles [22, 31]. A melting curve analysis was performed to exclude genomic DNA contamination and to confirm primer specificities and relative mRNA levels were calculated using the 2 ^−ΔΔCT^ method [57].

### Statistical analysis

Significance was analyzed using one-way analysis of variance (ANOVA) to test homogeneity of variances via Levene’s test, followed by Student’s t-test. Calculations and figures were plotted using Prism 7.0 (GraphPad Software Inc., San Diego, CA, USA). Differences were considered to be statistically significant for p-values < 0.05. Scale bars show the SEM of at least three separate experiments.

## Electronic Supplementary Material

Below is the link to the electronic supplementary material.

Supplementary Material 1: **Table S1**. Statistical summary for RNA sequencing results

Supplementary Material 2: **Table S2**. The value used for comparison between the results of gene expression levels of RNA-seq and qRT-PCR

Supplementary Material 3: **Table S3**. Diferentially expressed gene in normoxia and hypoxia

Supplementary Material 4: **Table S4**. Diferentially expressed gene between normoxia and hypoxia in TBCs and DLCs respectively

Supplementary Material 5: **Table S5**. Diferentially expressed gene in different incubation stages in TBCs and DLCs

Supplementary Material 6: **Table S6**. GO assignment for diferentially expressed gene in normoxia and hypoxia

Supplementary Material 7: **Table S7**. GO assignment for diferentially expressed gene between between normoxia and hypoxia in TBCs and DLCs respectively

Supplementary Material 8: **Table S8**. GO assignment for diferentially expressed gene in three different incubation stages in TBCs

Supplementary Material 9: **Table S9**. KEGG assignment for diferentially expressed gene in normoxia and hypoxia

Supplementary Material 10: **Table S10**. KEGG assignment for diferentially expressed gene between between normoxia and hypoxia in TBCs and DLCs respectively

Supplementary Material 11: **Table S11**. KEGG assignment for diferentially expressed gene in different incubation stages in TBCs

Supplementary Material 12: **Table S12**. Primer sequences used to amplify target genes by quantitative real-time polymerase chain reaction (qRT-PCR)

Supplementary Material 13: **Figure S1**. Validation expression levels of the eight randomly selected genes detected by the RNA-seq(n=3) and qRT- PCR(n=6). **Figure S2**. The top 20 of classification of gene ontology (GO) in three main categories and the top 15 pathways of DEGs on days 8, 12, and 18 of incubation between (A and D) NTBC8 and NDLC8, (B and E) NTBC12 and NDLC12, and (C and F) NTBC18 and NDLC18. **Figure S3**. Regulatory network of DEGs in the three incubation periods of days 8, 12, and 18 in DLCs under hypoxia and normoxia

## Data Availability

The datasets used and/or analysed during the current study are available within the article and its supplementary information files. All the raw sequences have been deposited in the NCBI database Sequence Read Archive with the accession BioProject number PRJNA940928.

## Abbreviations

DEGs: Differentially expressed genes
DLCs: Dwarf Laying Chickens
F6P: Fructose-6-phosphate
G6P: Glucose-6-phosphate
GO: Gene Ontology
TBCs: Tibetan chickens
