# Supplementary material for: Varied hypoxia adaptation patterns of embryonic brain at different development stages between Tibetan and Dwarf laying chickens
Source: BMC Genomics. 2023 Jun 21;24:342. doi: 10.1186/s12864-023-09457-4 (PMC10286358; doi:10.1186/s12864-023-09457-4)
Supplement: Supplementary file 13 — Supplementary Material 13: Figure S1. Validation expression levels of the eight randomly selected genes detected by the RNA-seq(n=3) and qRT- PCR(n=6). Figure S2. The top 20 of classification of gene ontology (GO) in three main categories and the top 15 pathways of DEGs on days 8, 12, and 18 of incubation between (A and D) NTBC8 and NDLC8, (B and E) NTBC12 and NDLC12, and (C and F) NTBC18 and NDLC18. Figure S3. Regulatory network of DEGs in the three incubation periods of days 8, 12, and 18 in DLCs under hypoxia and normoxia [file 12864_2023_9457_MOESM13_ESM.docx]

**Supplementary materials**

**
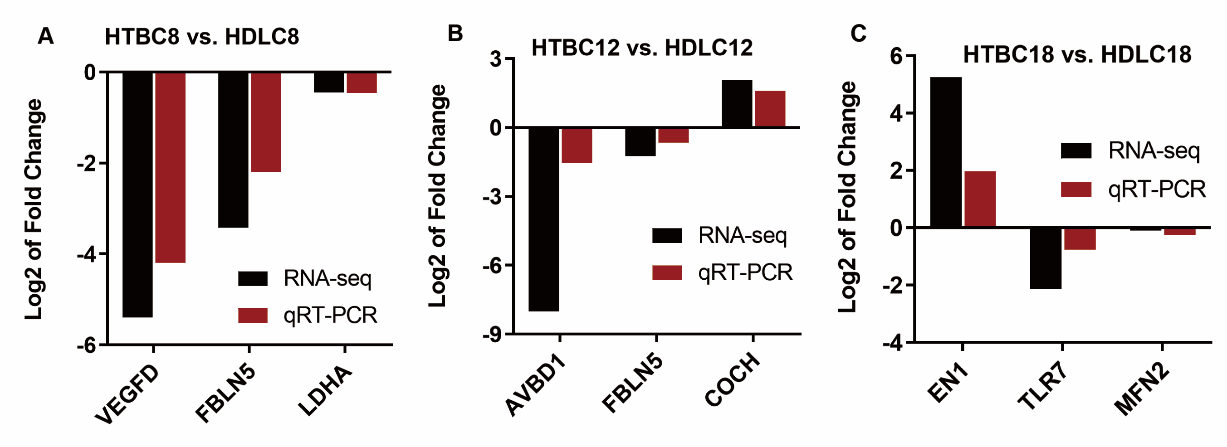
**

**Figure S1. Validation expression levels of the eight randomly selected genes detected by the RNA-seq(n=3) and qRT- PCR(n=6).** (A) The changed expression levels of selected genes between HTBC8 and HDLC8; (B) The changed expression levels of selected genes between HTBC12 and HDLC12; (C) The changed expression levels of selected genes between HTBC18 and HDLC18.


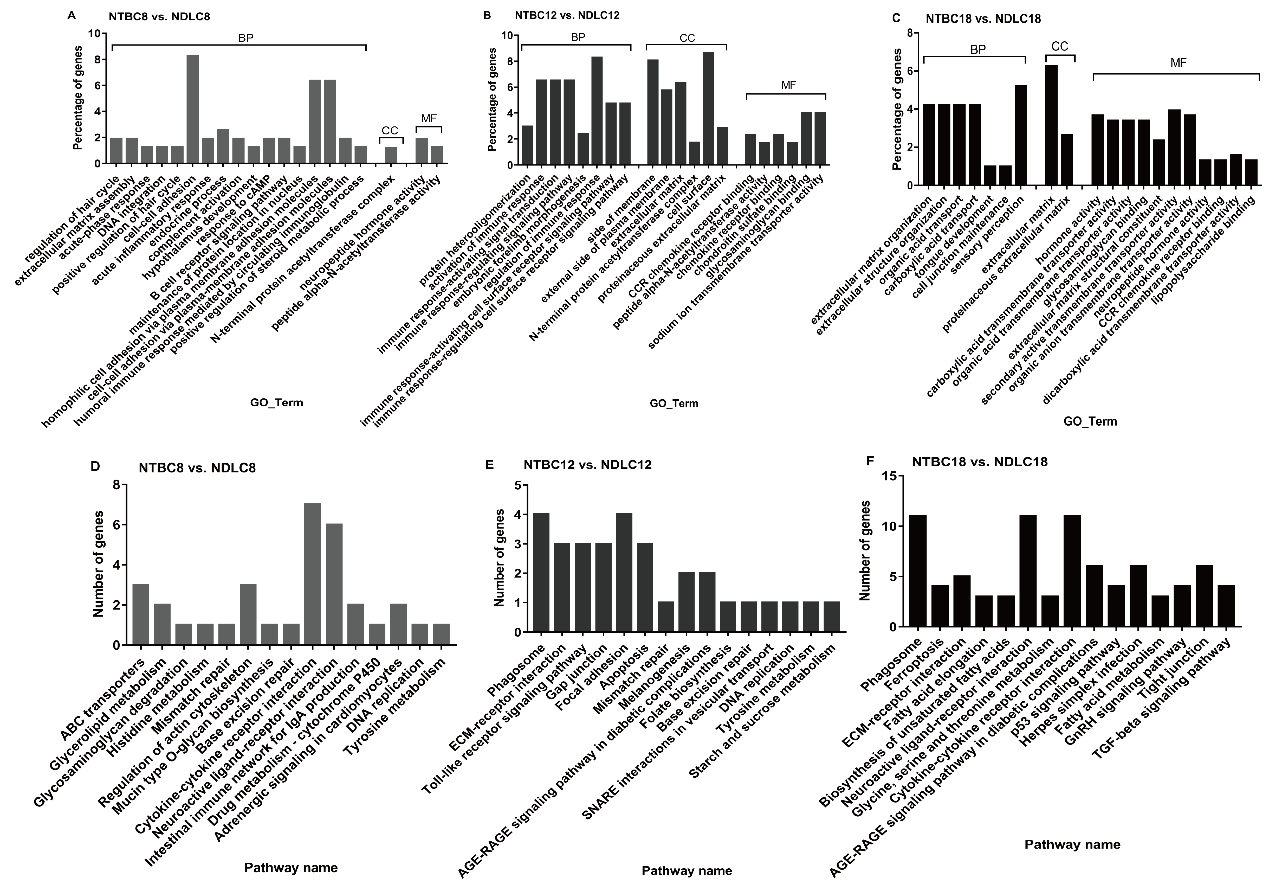


**Figure S2.** The top 20 of classification of gene ontology (GO) in three main categories and the top 15 pathways of DEGs on days 8, 12, and 18 of incubation between (A and D) NTBC8 and NDLC8, (B and E) NTBC12 and NDLC12, and (C and F) NTBC18 and NDLC18. The complete list of GO assignments can be found in the Supplementary Materials. (BP: biological process, CC: cellular component, and MF: molecular function)


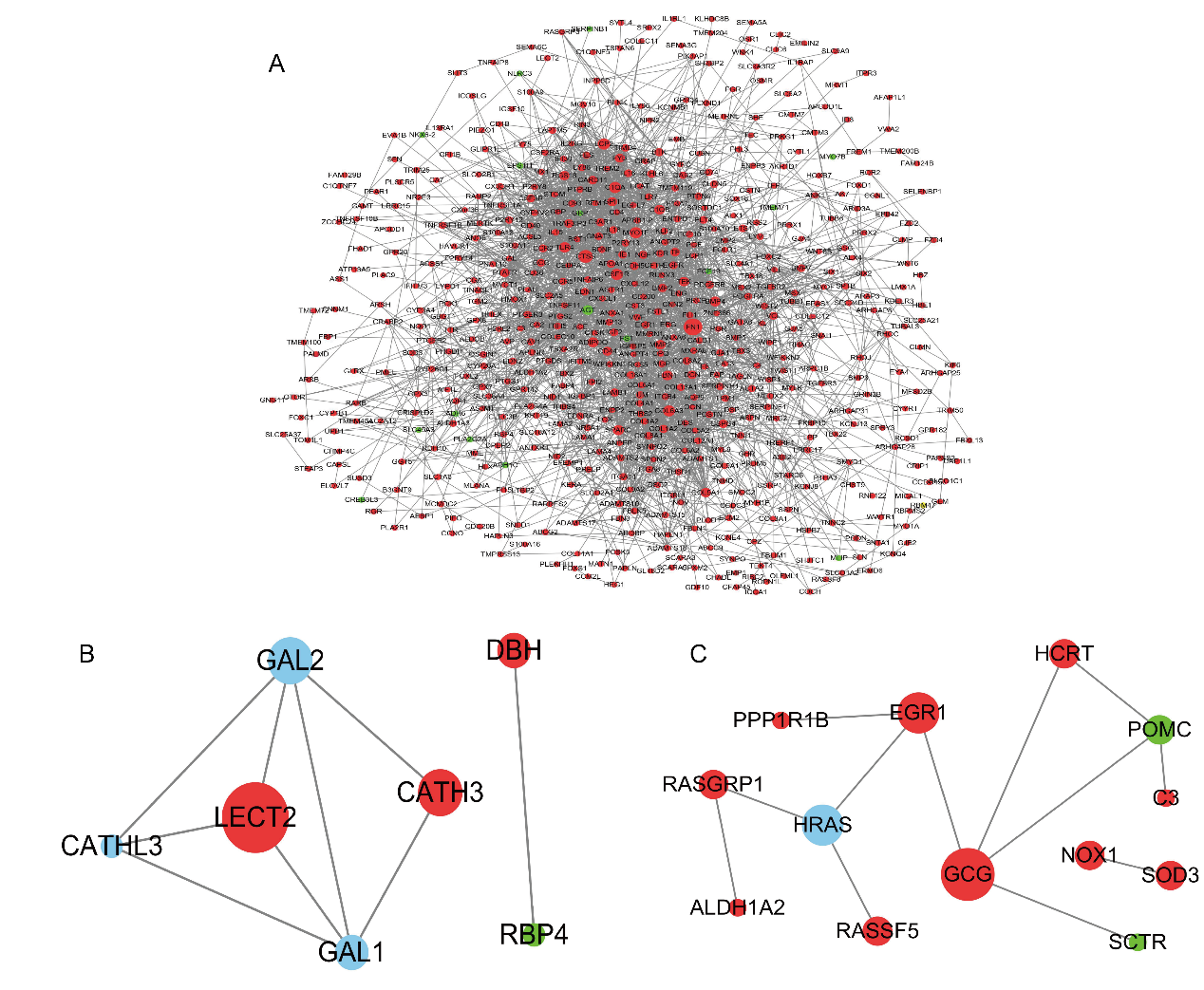


**Figure S3. Regulatory network of DEGs in the three incubation periods of days 8, 12, and 18 in DLCs under hypoxia and normoxia.** (A) Interaction network of DEGs between HDLC8 and NDLC8; (B) Interaction network of DEGs between HDLC12 and NDLC12; (C) Interaction network of DEGs between HDLC18 and NDLC18. Nodes (circles) represent the proteins encoded by DEGs. The radius of the circle indicates the significance of enrichment, red indicates that the expression of DEGs is relatively more abundant, green indicates that the expression of DEGs is relatively less abundant and blue indicates other. DEG, differentially expressed gene.
